# “Just a Typical Teenager”: The Social Ecology of “Normal Adolescence”—Insights from Diabetes Care

**DOI:** 10.1002/symb.42

**Published:** 2012-12-12

**Authors:** Davina Allen

**Affiliations:** Cardiff University

**Keywords:** accounts, vocabularies of motive, mothers, adolescence, diabetes

## Abstract

In Western society “normal adolescence” is understood to be a biologically driven phase characterized by emotional turmoil and irrational behavior. Despite being discredited within academic literature this discourse persists both in formal theory and everyday use. Drawing on the case of diabetes care, I argue that the discourse of “normal adolescence” derives its power from its value as a vocabulary of motive through which to navigate the contradictions inherent in the social order at this stage of the life-course. While helping us to comprehend sociologically the ecological niche in which “normal adolescence” is sustained, this analysis raises questions about the persistence of this discourse for social action.

## INTRODUCTION

In Western society, the lives of young people are widely interpreted through a discourse of “normal adolescence.” Adolescence is understood to be a biologically determined universal stage on the path to adulthood (Prout and James 1990) characterized by emotional turmoil and irrational behavior (Elliot 2010; Watson 2000; Webb, Jones, and Dodd 2001). While there are certain physiological and social realities that it is impossible to deny, scholarship has demonstrated that what is seen as “normal adolescence” is socially constructed. Yet empirical studies of young people and their families reveal that assumptions about “normal adolescence” are highly influential in shaping understanding of this stage of the life-course in formal theory (Macfarlane 1996) and folk wisdom (Alderson 1996; Elliot 2010). As Hacking (2004, 2007) has observed, “biologizing” and “normalizing” are central engines through which human sciences ‘make up people’, but they are sustained through their everyday use by ordinary actors. Understanding the persistence of particular categorizations of human kind therefore requires analysis of the social ecology which supports them.

Empirical studies of adolescence have shown the boundaries between childhood and adulthood to be complex, with social expectations of young people and their parents unclear. Drawing on the case of diabetes care, and symbolic interactionist analyses of how people account for their behavior, I argue that “normal adolescence” persists because it provides a “vocabulary of motive” (Mills 1940) through which to negotiate the moral contingencies that characterize the social order at this stage of the life-course. Through an examination of how mothers talk about their experiences of supporting their child to live a life with diabetes, I show that their accounts are oriented to certain behaviors as morally culpable and that they evoke taken-for-granted assumptions about “normal adolescence” to excuse their child's and justify their own actions. “Normal adolescence” can be understood as one element in a constellation of ideas about ‘a natural lifetime’ that includes childhood and adulthood and which is oriented to and reflexively constituted through everyday interaction. While appeals to “normal adolescence” provide sense-making resources with which to manage the contradictions that shape the transition from childhood to adulthood, as long as the actions of young people are understood as outside of individual control rather than as a rational response to a social problem then the normative order remains unchallenged.

## “NORMAL ADOLESCENCE,” YOUNG PEOPLE, MOTHERS, AND DIABETES

The origins of contemporary discourses of “normal adolescence” can be traced to nineteenth century developmental psychology and in particular the work of Granville Stanley Hall (1904). Hall established a number of core assumptions that have been highly influential in understanding this stage of the life-course (Simmons and Blyth 1988). First, adolescence as a transitional phase is believed to be a universal experience with its roots in human biology. Second, it is considered to be a period of “storm and stress” characterized by conflict with parents. Third, it assumes a “pre-social self” which exists within the individual but which must be found and developed. Fourth, it is seen as a period in which rational thought is not fully formed (France 2000). Difficulties exist with each of these assumptions. Anthropological and historical research has demonstrated that what it means to be young varies between cultures and over historical periods, although recent scholarship indicates such differences may not be as marked as once believed (O'Day 1994; Pollock 1983). The characterization of adolescence as a time of emotional turmoil has also been called into question, with an accumulative body of work revealing the conventionality of contemporary youth. For example, Gillies, Ribbens-McCarthy, and Holland (2001) studied “ordinary” young people and their parents living in a range of circumstances in the United Kingdom (UK) and found few identified with representations of the teenage years as particularly difficult. Moreover, anthropologists have shown that young people in less complex societies make a smooth transition to adulthood (Evans-Pritchard 1951). Concerns have also been raised about the claim that a pre-social self evolves into a full-fledged self during adolescence. It is argued that this overlooks the complexity of identity construction which is not related to physiological processes but arises out of negotiations between the individual and wider social context (James 1993; Jenks 1996). Finally, problems exist when ideas about rationality are explored. The psychological literature on youth assumes that rationality exists through processes of cognition. However, as Green (1997) has observed, rationality is a social construct and should be understood as an ideology rather than as descriptive of systems of thought.

Our ideas about “normal adolescence” may be socially constructed, but empirical studies of adolescence in the Western world reveal this stage of the life-course to be characterized by contradictory norms and values (Elliot 2010; Pascoe 2007; Spencer 2005). Holdsworth and Morgan (2005) describe it as “liminal state” in which young people and their parents are caught betwixt and between childhood and adulthood. The legal classifications that can be understood to define adult responsibility, such as the age at which young people can drink alcohol, earn money, leave full-time education, smoke cigarettes, join the armed forces, and consent to sexual relationships are incredibly varied (James, 1986). Expectations for parents are also uncertain. Defined as a process of letting go (Karp, Holmstrom, and Gray 2004), parents are expected to encourage independence but are still held to account by society for their child's behavior.

The experiences of young people with type 1 diabetes and their parents during the transition from children's to adult services represent an interesting microcosm of these tensions. Living a life with diabetes depends on the individual injecting insulin several times daily or making regular adjustments to the continuous rate of insulin infusion through a pump-delivery system, monitoring blood glucose levels, and attending closely to diet and exercise. The complex nature of diabetes self-care makes it difficult to manage well and most of them require assistance to live a life unconstrained by the condition (Corbin and Strauss 1988). In line with normative gender expectations, in childhood this role is typically performed by mothers (Allen et al. 2011; Williams 2002) but during adolescence approaches to care are designed to foster independence (Department of Health 2001). As the young person makes this transition, medical consultations become progressively focused on the young person rather than the parent and health professionals increasingly treat young people as theoretic actors; that is, capable of rational thought and able to choose between alternative courses of action (Silverman 1987). Once the young person has been defined as an active decision maker, they gain autonomy at the cost of being morally responsible for their actions and are accountable when a gap exists between their knowledge of the parameters of good management and actual behaviors. As Williams (2002) has observed, this process places mothers in a no-win situation. On the one hand, they are expected to support their child's progression to independent management and are perceived as over-protective if they do not. On the other hand, they feel responsible for their children's behavior when things go wrong (Ribbens 1994).

## MOTIVATIONAL ACCOUNTS

In this paper, I examine how mothers talk about their experiences of supporting a child to live a life with diabetes showing how they appeal to “normal adolescence” to account for behaviors that fall short of the recommendations of health professionals. As such, this paper builds on a longstanding interest in symbolic interactionism, arising from the pragmatism of Dewey (1922) and Burke (1935), with how actors legitimize their behavior and manage identity (see Albas and Albas 2003 for a comprehensive history). Mills (1940), the first sociologist to elaborate these ideas, asserted that people draw on a “vocabulary of motives” to answer for their actions in a contextually appropriate manner. Mills was at pains to stress that vocabularies of motive follow rather than determine action. Building on these insights, as Albas and Albas (2003) point out, Scott and Lyman (1968) replaced the term motive with account to make this relationship clearer. Accounts presuppose that individuals socially negotiate the meaning of events and can change them by reconfiguring their underlying meaning. They are born out of the distinctly human capacity to be blamed, charged, and held responsible (Järvinen 2001). They are a crucial element in the social order because they prevent conflicts from arising by discursively managing gaps between action and expectation.

Studies of accounts have developed along two lines (Davis 2000). They have been examined as a way to neutralize deviant behavior and respond to challenges of some kind (Scott and Lyman 1968). Here their function is “to shore up the timbers of fractured sociation, to throw bridges between the promised and the performed, to repair the broken and restore the estranged” (Scott and Lyman 1968:46). In Goffman's (1971) terms, they are a type of “remedial work” which enables actors to manage their self-presentation and “maintain face.” Accounts have also been examined more generally (Davis 2005), as “explanatory self narratives” that allow people to “create and organize meaning” as well as define and manage identity (Davis 2000:35, 38, cited by Estes 2011). Here accounts can be preemptive (Hewitt and Stokes 1975; Kolb 2011) or anticipatory (Murphy 2004) rather than reactive. Davis has made the case for drawing together the respective emphases of each tradition to advance understanding in this field, although in practice there is much implicit overlap in the approaches that scholars have adopted (see Estes 2011 for an explicit example).

Considered in these broad terms, the concept of accounts has been widely used to analyze peoples' explanations for a variety of behaviors ranging from murder (Ray and Simmons 1987), sex offenses (Taylor 1972; Scully and Marolla 1984; Higginson 1999) and criminality (Harris 2011), through gambling (Rossol 2001; Smith and Preston 1984), homelessness (Snow and Anderson 1987) and alcoholism (Järvinen 2001), to student absenteeism (Kalab 1987), legitimating the first tattoo (Irwin 2001), and work conduct (Kolb 2011; Shulman 2000). In the sociology of health and illness it has been deployed *inter alia* in the analysis of mothers' defense of their infant feeding decisions (Murphy 1999), body builders' legitimation of steroid use (Monaghan 2002), the negotiation of risk behaviors by HIV positive people (Rhodes and Cusick 2002), and management of mobility in arthritis (Rosenfeld and Faircloth 2004). In studies of parenting, it has been used to explore how parents accomplish moral adequacy (Baruch 1981), account for teen sexuality (Elliot 2010), and respond to their homosexual sons and lesbian daughters coming out (Fields 2001).

For current purposes, the importance of studying accounts is that they provide important clues about the culture in which the individual is enmeshed (Taylor 1972). Accounts are intrinsically social and the utterances that constitute a satisfactory motive are circumscribed by the situation (Mills 1940). Along with rules and norms of action for various situations, we learn vocabularies of motive appropriate to them and, as number of studies have shown, available motivational accounts are shaped by social positioning (Higginson 1999; Kolb 2011). As Mills (1940) observed, the different reasons actors offer for their actions are not themselves without reasons.

## DATA AND METHODS

This paper analyses qualitative data generated as part of a realistic evaluation (Pawson and Tilley 1997) of the transition from child to adult diabetes services in the UK (Allen et al. 2012). The study was designed to ascertain what works best to support transition, for whom and in what circumstances. Five service models were purposively selected to reflect the spectrum of provision and longitudinal case studies of young people with type 1 diabetes undergoing transition were undertaken in each. Potential cases were identified by appropriately placed healthcare professionals and a purposive sample selected. The sampling strategy was designed to ensure representation across the full transition process and was stratified by gender. Once the young people had agreed to take part, they were asked to identify their principal carer and all identified their mother as fulfilling this role (Table 1).

**TABLE 1:** Case Study Participants

Transition Service	Females	Males	Carers
Service 1	1	4	5
Service 2	2	5	6
Service 3	5	4	7
Service 4	8	3	10
Service 5	7	7	11
Total	23	23	39

Interviews with young people and their mothers were undertaken at three time points over 12–18 months. The first and third were face-to-face and lasted approximately an hour and the second was by telephone and lasted approximately 30 minutes. Face-to-face interviews were carried out mainly in the young person's home, with a minority undertaken in alternative venues such as cafes. Most were carried out separately and in private; although in a small number of cases at the request of the family the young person or parent was present at the time of the other party's interview. National Health Service Research and Development Office and Research Ethics Committee approvals were received. Interviews were transcribed in full and edited to remove identifying materials. Analysis was supported by the use of computerized qualitative data analysis software: Altas/ti.

In this paper, I draw on the 117 interviews undertaken with the 39 mothers participating in the study. These explored mothers' experiences of caring for a child with diabetes, perceptions of the service, the process of negotiating independence and significant life-course events. In the original study interviews were treated as a resource from which to evaluate approaches to transition. This analysis revealed that mothers frequently appealed to ideas about “normal adolescence” in their sense-making. In this paper, the interviews are treated as a *topic* in which these references to “normal adolescence” are scrutinized in greater detail for the interactional work they perform and for what they reveal about the social order.

All healthcare practices take place within a moral framework (Parsons 1951) and there is a perceived need for young people with diabetes and their parents to show they are doing their best to manage the condition. Moreover, interviews are “identity occasions” (Holstein and Gubrium 1995) and subjects are working to produce themselves as competent actors in the eyes of the interviewer. Mothers were aware that their child and healthcare providers would also be interviewed and that the young person's clinical record would be consulted. Therefore, any actions which deviated from medically prescribed treatment plans would be revealed, precipitating participants to orient to any perceived misalignment of behavior and expectation and accommodate this in the course of their interaction with interviewers. In Western society, mothers are positioned as mediators between their child and others (New and David 1985) and in describing their experiences of supporting their child's diabetes management the mothers in this study were engaged in two kinds of identity work: management of their self-presentation and management of the presentation of their child.

Scott and Lyman (1968) identify two kinds of accounts: excuses and justifications. To offer an excuse is to appeal to approved vocabularies for relieving responsibility when it is perceived that conduct may be questioned. To offer a justification is to give an account in which responsibility is accepted for the act in question, but the negative qualities associated with it are denied. In the second part of this paper, I wish to show how mothers' accounts of parenting a young person with diabetes are oriented to certain behaviors as morally culpable and appeal to “normal adolescence” to *excuse* their child's and *justify* their own actions. In this context “normal adolescence” can be understood as a vocabulary of motive (Mills 1940) through which social conduct can be interpreted and which can be deployed as an exculpatory resource in making sense of behaviors and managing identity at this stage of the life-course.

## JUST A TYPICAL TEENAGER

References to “normal adolescence” were a predominant feature of mothers' accounts.

[B]eing an adolescent anyway without diabetes is really difficult, they go through that phase they're not very communicative […] they don't want to share things, you might say something and that's it they go defensive.

[1-M148]

[S]he can be a quite stroppy teenager at the moment so we have the usual conflicts.

[5-M62]

[T]hrough these teenage years, they're quite sort of hormonal.

[4-M45]

Common to all these extracts is the assumption that adolescence is a universal phase characterized by conflict and moodiness caused by hormones. This constellation of attributes is treated as a matter of natural fact, with each mother making reference to what everyone knows about the life of young people. Indeed, in the second extract, the mother's talk moves ineluctably between a description of her own child's behavior to that of young people in general: “we have the usual conflicts.” Such ideas were also reinforced by health professionals. The following excerpt is from a textbook on adolescent medicine.

“Adolescence is the space of life […] between childhood and maturity. It is a time when the body reaches physical perfection but it is also a time when, as Keats wrote as a 23 year old in Endymion: ‘the soul is in ferment, the character undecided, the way of life uncertain, the ambition thickset’”

(Turnberg 1996:iii)

Of course doctors generalize about adolescents from a biased sample insofar as they mostly see the ones with ‘problems’ but they still get treated as authorities.

[T]hey warned us that a lot of them (young people) do just go completely off the rails and don't be surprised if she stops testing and don't be surprised if.

[5-M63]

[W]e were told that everyone rebels, anyone that you know, is young, they will have a time of rebellion where they'll think I can do what I want, I'm going to eat what I want, you know, I don't care you know, nothing will happen.

[4-M69]

So taken for granted was this constellation of ideas about the typical teenager that it was frequently offered as a proxy for a description of actual health behaviors. For example, in the following extract, in response to the researcher's question about her confidence in her daughter's self-management, Carol's mother offers the observation that “she's a teenager basically.”

R: And what about your confidence in her management and her confidence in her management?

M: She's a teenager basically, she's okay but she's a bit lax with everything […] she's doing okay but you know they just, if they think they can get away with it they will won't they.

[5-M63]

By deploying the notion of the typical teenager as a description of behaviors, this mother appeals to socially distributed stocks of knowledge about “normal adolescence” to account for her daughter being “a bit lax with everything.” Drawing the interviewer into this shared view of what everyone knows about teenagers, she argues that this laxity is understandable because “if they think they can get away with it they will won't they.”^1^ Her daughter's self-management might be less than perfect, but this is presented as normal for a young person at this stage of the life-course. In the extracts below, Heath and Rebecca's mothers make similar appeals in explaining the need for continuing involvement in their care and, once again, move from observations about their child's behavior to statements about those of teenagers in general.

Just prompting mainly, obviously for adolescents I think that's the, time-keeping on his behalf and things like that.

[4-M91]

I mean we do remind her, now and again we say like you know, how many blood tests have you done today and I know like you know, as they get older because she's like a teenager and […] they're in and out and they do forget, you know.

[4-M69]

Age implies social and moral obligations and it is common to draw on categories of aging to place people's identities and interpret their behavior (Atkinson 1980; Hockey and James 2003). Of significance for current purposes is the observation that the corporeality of aging renders it ripe for biological determinism. As an explanation of human behavior, biologizing has been a regular focus of sociological critiques, but here it is deployed as an exculpatory resource. In appealing to their child's status as a teenager to account for suboptimal health behaviors, mothers offered a category of excuse Scott and Lyman (1968) call fatalistic forces. Scott and Lyman argue that in various cultures fatalistic forces are considered to control some or all events and that biological drives are among the fatalistic items most likely to be evoked, with the body and its physiology attributed with influencing behaviors for which actors may wish to relieve themselves of responsibility. In this context, “normal adolescence” provides a socially acceptable excuse with which to account for the gap between medically-determined treatment plans and the young person's actual behaviors. They also enable mothers to do another kind of identity work in circumstances in which concealment was not possible. By acknowledging the short-comings of their child's self-management practices mothers are simultaneously establishing themselves as knowledgeable actors who have an awareness of the recommendations of health professionals thereby demonstrating their own moral credibility (Baruch 1981).

Appeals to “normal adolescence” also provide mothers with access to another mechanism for neutralizing blame: defeasibility. Defeasibility is a category of excuse that addresses the widespread view that all action entails a theoretic element. Thus untoward actions can be rebutted on the grounds that an actor was not fully informed or that their will was not completely free. Young people with diabetes and their families receive volumes of information about management of the condition so there is little scope to claim that poor management arises from faulty knowledge. However, in addition to appealing to their biology, discourses of “normal adolescence” also enable mothers to evoke common stocks of knowledge about the forces of peer pressure at this stage of the life-course. Here their accounts portray the young person as more acted upon than acting. In the following extract Hayley's mother is recounting her daughter's several admissions to hospital since the last interview.

Yes she's been in hospital a couple of times, mainly I think caused by drinking, not excessively but I think she's at the age where a lot of the teenagers who want to be, as Hayley put it, “normal.”

[1-M82]

In this case, the hospital admissions were all alcohol-related, but in accounting for Hayley's behavior her mother emphasizes that she was not doing anything different from any other normal teenager and so should not be condemned for behaving like her peers. Alex's mother marshals a similar claim in describing her son having tried alcohol.

I mean he doesn't drink, I think he's tried things but more because of peer pressure and I think he's decided not to you know, I know he shouldn't be but they do and he's decided not to.

[5-M41]

As before, an interesting feature of both these extracts is the interactional work undertaken by mothers to distance themselves from their child's behavior and display their own moral credibility, revealing the dual identity work that is being accomplished in these accounts. In the first extract ‘normal’ is put into reported speech by Hayley's mother suggesting that although her daughter may be drinking, her mother does not endorse this view. Alex's mother also explicitly observes that she knows he should not be drinking.

Such appeals derive their power from the positive social value accorded to being ordinary in Western society (Sacks 1985). Diabetes management, like other chronic conditions, is framed within discourses of normalization, with young people and their families encouraged by health professionals to live a life un-constrained by the condition. In descriptions of their experiences of coping with diabetes throughout childhood only a small number of respondents referred to curtailing their activities; more often, both young people and mothers offered examples of events in which they had participated unencumbered by the condition. These were typically described in some detail as celebratory tales that illustrated the family's success in adapting to the diagnosis. When young people enter adolescence, however, this emphasis on normalization creates strains because the social world of teenagers is presumed by health professionals to be fundamentally hazardous (Macfarlane 1996). By evoking their child's normality or typicality in their accounts of their behavior, mothers can manage these tensions by drawing on the positive aspects of one element (normality) to diffuse the potential damage to self associated with the other (risk behaviors). There are parallels here with Estes' (2011) analysis of mothers' and fathers' management of the student-parent dilemma in higher education in which respondents joined their identities as students and parents by describing their education as beneficial to their children and their children as advantageous to their education. These accounts “neutralize negative evaluations” related to combining parenthood and higher education, serving to defend their identities as parents and students.

A further indicator of the dominance of the discourse of “normal adolescence” is that behaviors not in alignment with societal expectations of youth were also accountable. Mothers of young people not perceived to lead full social lives or who had chosen not to experiment with so-called risk behaviors, were moved to explain this ‘abnormal’ behavior. In the following extract, Alex's mother describes her son as sensible relative to his friends but is at pains to stress that he accomplishes this without being “geeky” and “boring.”

I actually think Alex is quite a sensible boy and whether it's because of his diabetes I don't know but compared with some of his other friends he's actually, what he says about things like what teenagers get up to, he's very much not like that and although he wants to be with them and everything, things like trying alcohol and things like that, he's quite vehemently against and manages to be not one of the geeky boring people but kind of blends in with them.

[5-M41]

Similarly, in explaining that her daughter does not go out much in the following extract, the mother emphasizes that this is because “a great big party scene” does not exist. Of particular interest is her repair in the third line of this extract in which she appears to be about to attribute her daughter's lack of partying to personal qualities (“I mean she is not”) and redirects her account to external causes: “there's not this great big party scene.”

I mean generally speaking she's not a teenager that goes out that much, like I say she goes out with her friends into town and they're always going to Starbucks but I mean she's not, there's not this great big party scene or anything.

[4-C52]

## MORALLY ADEQUATE MOTHERHOOD

Having examined how “normal adolescence” enables mothers to excuse their child's suboptimal self-management, I wish now to consider how they account for their own behaviors. Whereas discourses of “normal adolescence” are called upon to *excuse* the health behaviors of their children; mothers made similar appeals in order to *justify* their own actions. Like excuses, justifications are socially approved vocabularies that neutralize an act or its consequences when these are called into question. However, as Scott and Lyman (1968) argue, there is a crucial difference. To justify an act is to assert its positive value in the face of a claim to the contrary. Justifications recognize that the act in question was not permissible, but claim that the circumstances allow for it. The neutralization technique most evident in mothers' accounts was an appeal to the idea that adolescence can be a time of storm and stress and that parental support for the young person's diabetes management has to be balanced against the risks that too much interference may cause them to rebel and disengage entirely. This was evoked by mothers who were accounting for occasions in which they perceived that they could be accused of acting irresponsibly by not managing their child sufficiently. In the following extract, the mother is talking about a common problem: encouraging young people to take regular measurements of blood glucose levels. Here she describes at some length the demands of this process and the challenges of sustaining this in the context of family life. It is clear, however, that she considers that such practical considerations might be seen as insufficient grounds for any perceived deficiencies on her part; hence her ultimate justification is that excessive interference might lead her child to rebel.

[The doctor] doesn't take any crap from me, or from her, you know, he will say yes, yes, if I say well we asked her to do a reading but she didn't do a reading, well, you know, thinking where's the parental control, you make sure that they do the reading. And he's right but it's not always that easy so you know that she needs to do a reading in the morning and a reading at lunch and a reading at tea time and a reading before she goes to bed or something and you might have had one day and say you've done it in a morning and she's felt okay and you know they've got enough finger pricks in their hands and you think oh yes she's looked okay and you go a bit by how many times she goes to the loo ((washroom)) and everything else but you might be doing something else as well or you've got to take one to an evening thing, got to pick the other one up, and you think oh yes she's alright, well do your finger prick, oh I'm alright but you don't do it and so then when you go and they say oh she's actually not done her readings all the time and you're thinking oh I suppose I should be a bit stricter. But you don't want them to rebel.

[4-M41]

Kate's mother makes a similar kind of appeal to account for her claim that when food goes missing in the family home she does not make an issue of it.

On the whole she manages really well […] but sometimes she can be a bit rebellious and secretive, you know, there are packets of things that go missing that nobody's had until they turn up under Kate's bed or something and I don't generally make too much of it, I nag a bit but I don't make too much of it because I think at her age, if we make it a real issue, she'll be even more determined to do it.

[4-M45]

In both these excerpts, mothers build a case that they are permitting a lesser evil in order to ensure their child's continued engagement with their self-management. Here “normal adolescence” furnishes mothers with a neutralization technique, not previously identified in the literature, that I will call “an appeal to higher order values.” In such instances the actor asserts that their action was permissible or even right since it served the interests of an over-riding value. In the examples considered here, mothers account for their behaviors by reference to concern for their relationship with their child and ensuring they remain engaged with the management of their condition. As such they are evoking socially distributed knowledge about adolescence being a time of emotional turmoil and, in the context of diabetes care, recognition of the risk of young people disengaging from the service (Department of Health 2001). Such appeals also orient to “normal adolescence” as a transient developmental stage in which a suboptimal situation may be temporarily tolerated.

## BRACKETING THE PRESENT AND CONSTITUTING FUTURES

The mothers in this study regarded independence to be a positive social value, and acknowledged that achieving this should be an incremental process of handing over responsibility for diabetes care (Williams 2002). However, they also described this process as difficult, particularly if they perceived their child was not managing the condition as well as when it was under greater parental control. Many described the need to adjust to their reduced role and pointed to the re-alignment of responsibilities that this entailed (Allen et al. 2011). Accounts displace lived experience to some extent, but this should not distract from their existential basis (Rhodes and Cusick 2002). Beyond their function in the interactional management of untoward behaviors, discourses of “normal adolescence” also facilitate the bracketing off of difficulties in the present, while simultaneously constituting a more positive future. As Sykes and Matza (1957) observe, techniques of neutralization may be insufficient to shield the individual from the impact of internalized values and many mothers expressed fear and concern about the long-term consequences of their child's failure to manage their condition. In this context, “normal adolescence” furnishes an additional sense-making resource which further explains its ubiquity: the belief that adolescence is a transient phase. A recurrent feature of mothers' accounts of current difficulties was their representation as a temporary state of affairs from which their child would eventually emerge.

You're watching your own child destroy all the good work you've done basically and I guess I just try and remain optimistic that he will come out the other side and get back on track again.

[4-M16]

[H]opefully there will come a point when […] he's a bit more sensible about it.

[3-M93]

Because I think with the lifestyle that he's got at the moment he's not doing the diabetes any good but I think that he's at a moment in his life and I think he will come out the other end.

[3-M94]

If present difficulties can be accounted for by recourse to “normal adolescence,” they leave in play a more optimistic future in which the fatalistic forces of biology will abate and responsible adult behavior adopted. We can see here evidence of the notion of a pre-social self prevalent in psychological models which assume adolescence to be a period during which the self is in the making and adult identities are being formed. Behaviors which fall short of the rational action considered the hallmark of adulthood can be bracketed off and excused as an expression of “normal adolescence” and outside the control of their child thereby neutralizing threats to self. There are clear parallels here with Harris' (2011) study of the identity work undertaken by offenders in which their accounts provide a way for people to distance themselves from their past in an attempt to re-create a possible future self.

## UNDERSTANDING THE SOCIAL ECOLOGY WHICH SUSTAINS “NORMAL ADOLESCENCE”

Thus far, I have shown how mothers appeal to “normal adolescence” in accounting for their own and their child's health behaviors. Understood as a transient stage and founded on the twin engines of normalizing and biologizing, “normal adolescence” furnishes mothers with a vocabulary of motive through which to excuse the imperfect health behaviors of young people, demonstrate their own moral adequacy and justify parental permissiveness in overseeing self-management. I have argued that the case of transition in diabetes management may be considered a microcosm of this stage of the life-course in which the boundaries between childhood and adulthood are uncertain and the expectations for young people and their parents unclear. In the final part of this paper I want to move beyond the specific case of diabetes to the status of young people in general and the social ecology through which “normal adolescence” is sustained. Having identified the interactional work that “normal adolescence” is being deployed to accomplish, I look backward to consider what features of the social order this presupposes, the processes through which this is constructed and the consequences of the dominance of discourses of “normal adolescence” as motivational accounts for enabling and constraining action.

“Normal adolescence” can be understood as one element in a constellation of ideas about ‘a natural lifetime’ which begins at birth and ends in death and which forms part of the basic conceptual equipment through which everyday activities are organized (Atkinson 1980). Atkinson (1980) argues that in-so-far-as members treat this ‘natural lifetime’ as a fact, then the sociologist's task is to locate the practical reasoning through which this is accomplished and how actors use that version to reflexively constitute social settings as they conduct their everyday affairs. This entails the scrutiny of the interactional work through which social actors make themselves and others observable-reportable as a ‘child’ or an ‘adult’ or as ‘growing up’. Of interest is how particular ‘lifetime’ categories are selected and used to decide the sense of actual talk and activities. He argues that actors treat the ‘natural lifetime’ as a normative order that, in the words of Zimmerman and Pollner, is: “pre-supposed by members as an enforceable schema of interpretation and guide to action that is used by members to present themselves in a particular fashion and witness the talk and conduct of others in stable ways” (1972:87). In order for actors to produce the facticity of a natural lifetime in their talk it is necessary for them to attach descriptors that are taken to be tied to different stages in the lifetime schema. Drawing on Sacks' (1972) notion of membership categorization devices, he argues that the categories comprising the lifetime collection are: child, adolescent, and adult. This collection has several properties. First, they are staged—a lifetime is a unidirectional progression. Any single person can be categorized first as a child, second as an adolescent, and third as an adult. Second, no person can at any one point be assigned to more than one category from the lifetime collection. Third, certain activities which members might treat as tied to any one of these categories may not be tied to other categories from the lifetime collection. He suggests that in everyday talk actors orient to particular lifetime categories as ‘staged’ and it is through this orientation to particular categories as staged in conjunction with their treatment of particular activities as being tied to particular membership categories that growing up or failure to do so in a proper manner is made observable.

Atkinson restricts his analysis to the membership categories of ‘child’ and ‘adult’ and falls short of specifying the attributes tied to particular lifetime categories. He is more concerned with explicating the rules through which the facticity of a natural lifetime is accomplished. However, observing and reporting are moral activities in that one has to continuously select descriptors that are hearable as sensible, right and appropriate and a particular description has an occasioned rightness (Atkinson 1980, Sacks 1985). As Sacks (1985) has put it, ‘doing being ordinary’ takes work and effort and requires knowledge of what everybody does *ordinarily*.

In this paper as we have seen, mothers and health professionals treat certain constellations of attributes as natural facts of “normal adolescence.” Following Gubrium and Holstein (1990), I suggest that these can be considered as a ‘configuration of concern’ which draw together and convey what everyone knows about the attributes that characterize this stage of the life-course. A configuration of concern is a pattern of collective representations (Durkheim 1938) which links sentiments and ideas so that “as we borrow one term to describe experience, we are urged to borrow from the rest.” (Gubrium and Holstein 1990: 152). Gubrium and Holstein apply this framework to the analysis of family discourse and show how this configuration tacitly links “household,” “family,” “privacy,” “house,” “home.” These authors emphasize that the configuration does not inform us how these connections should be made: “application is *guided* by the configuration, not *determined* by it” (1990:153 original emphasis). This, they suggest, enables the family to be talked into diverse forms in different institutional contexts.

What is striking about the data in this study, however, is the remarkable regularity with which the young person's actions are portrayed as outside their control. There were no instances of untoward behavior accounted for by appeals to different kinds of vocabulary of motive centered on intentional action. There are parallels here with the situation of sex offenders described by Taylor (1972). Taylor examined the range of motivational accounts available to sex offenders, the role of others in determining which are principally acceptable, the variables which restrict the acceptability of alternative vocabularies of motive and the significance of alternative vocabularies for self-conception and future behavior. Ninety-four accounts offered for sexual deviancy from a range of sources were studied. He reports an overall limited repertoire of motivational accounts, with those which pointed to factors outside the control of the individual as most prevalent and most likely to be considered socially acceptable. As Taylor observes, Scott and Lyman (1968), unlike Mills (1940), do not concern themselves with the significance of accounts for action and the differential power of social institutions to impose a definition on the situation. He argues that other deviant groups historically accorded similarly limited vocabularies of motive which evoke external forces have been able to move toward more volitional accounts, but sex offenders appear to be locked into deterministic formats which always deny their behavior as a chosen solution to a problem. Adolescents appear to face the same constraints. Why should this be? Taylor suggests that in addressing this question attention should be directed at the benefits that arise for others from this state of affairs. This takes us toward the heart of the question posed at the outset of this paper about the features of the ecological niche through which “normal adolescence” is sustained.

As I have argued, our ideas about “normal adolescence” arise from a wider interpretative schema of a natural lifetime which assumes that there are clear differences between child (undeveloped), adolescent (partly developed), and adult (informed, mature, and dependable) (Alderson 1996; Atkinson 1980). This representation advocates a normative order as much as it conveys particular state of empirical reality, but this does not undermine its power as a source of social control. Young people are valued by adult society primarily because they are adults in the making (Frankenberg 1993) and adulthood is understood as tied to the attributes of rationality and responsibility. During the transition from childhood to adulthood, young people and their families must negotiate a range of contradictory expectations against which actual behaviors will often fall short. Persons adopting different versions of reality can be treated as doing so legitimately in so far as they are seeable as either “children” or “adolescents” (Atkinson 1980). In this context “normal adolescence” can be understood as a necessary counterpart to “normal adulthood” enabling young people and their families to negotiate this stage of the life-course in ways which are recognizably ordinary while sustaining the adult version of the world against which their behaviors are assessed. By projecting responsible adulthood as the desired end state, discourses of “normal adolescence,” bracket off and contain current troubles whilst upholding the aspiration that future behavior will fall into alignment with adult definitions of the normative order.

While “normal adolescence” provides an exculpatory resource in accounting for behaviors unacceptable to adult society, it is not without its social consequences. Discourses can position people, as well as people positioning themselves, and these positions can potentially limit their thoughts and actions (Parry et al. 2006). As long as the behaviors of young people are always accountable by reference to their biology, they can exist within the dominant social order, but ultimately this remains unchanged. The dominance of “normal adolescence” means that the focus of concern is directed at the level of the individual rather than to wider social, political, or economic constraints which might impact on young people's ability to successfully negotiate this stage of the life-course. Furthermore, “normal adolescence” has a self-reinforcing quality. Constituted through the definitional privilege of adulthood, any challenge to the normative order can be interpreted as evidence of hormonally driven rebelliousness rather than a legitimate critique thereby preserving the generational order.^2^ As Taylor (1972) has observed, once an individual can be allowed consciousness and can be considered to be making a decision to act in a particular way, then there is a possibility of revising our world view and for this behavior to be presented as a reaction to a special problem with social rather than individual origins.

## CONCLUSION

In this paper, I have argued that societal understanding of the lives of young people is dominated by a discourse of “normal adolescence.” The teenage years may be marked by certain indisputable biological and social realities, but what is widely understood as “normal adolescence” is socially constructed. Despite being discredited in the academic literature, ideas about adolescence as a universal biologically driven phase characterized by emotional turmoil and irrational behavior, prevail both in formal theory and everyday practice. My purpose here was to understand the ecological niche that sustains them. Taking mothers' accounts of diabetes management during adolescence as a case study, this analysis reveals that “normal adolescence” affords a vocabulary of motive with which to navigate the contradictions and tensions which characterize the social order at this stage of the life-course. Reflexively constituted through everyday interaction, “normal adolescence” can be understood as one element in a constellation of ideas about ‘a natural lifetime’ that includes childhood and adulthood and which forms a cornerstone of our sense of social order. In this sense then, “normal adolescence” is a necessary complement to ‘normal adulthood’. Nevertheless, it is also the case that such ideas afford a limited repertoire of motivational accounts for young people and those who speak for them. This has important social consequences; because as long as the behavior of young people is understood as outside of their control rather than as a rational response to a social problem then the normative order remains unchallenged and alternative formulations of the issues are difficult. The corollary of this is societal neglect of the specific needs of this group and a failure to address social arrangements which impact negatively on their lives.

## Notes

1 As Järvinen (2001) has shown, in accepting the accounts offered, interviewers cooperate in respondents' identity work.

2 There are parallels here with Field's study of the destigmatisising identity work of parents of lesbian women and gay men, which drew on normative understandings of gender, sexuality, and parenting to normalize their children and normalize themselves, but at the same time reinforced the gender, sexual, and familial norms that rendered them deviant in the first place.
